# Assessing whole-host homogenisation as a new tool for parasite detection and identification

**DOI:** 10.1016/j.crpvbd.2026.100348

**Published:** 2026-01-01

**Authors:** Kamil Hupało, Celine Sassor, Mia Merle Fuhren, Dominik Buchner, Daniel Grabner, Bernd Sures

**Affiliations:** a Department of Aquatic Ecology, University of Duisburg-Essen, Essen, Germany; b Centre for Water and Environmental Research, University of Duisburg-Essen, Essen, Germany; c Aquatic Ecosystem Research, University of Duisburg‐Essen, Essen, Germany; d Research Center One Health Ruhr of the University Alliance Ruhr, University of Duisburg-Essen, Essen, Germany

**Keywords:** Helminths, Eel, *Anguilla*, DNA-based species identification

## Abstract

Despite their importance in ecosystem functioning, parasites remain the most neglected components of biodiversity monitoring. This neglect is partly due to the methodological challenges associated with their detection and identification. Current traditional morphological and molecular approaches are time-consuming, labour-intensive, and require specialised expertise. Here, we explore a novel approach of deriving parasite information through whole-host homogenisation followed by DNA-based identification of its parasite biota. Our goal was to validate whether the approach is feasible and if it could become a complementary method for studying parasites, providing a time-efficient solution allowing for a holistic parasite scan with limited taxonomic expertise. To test the method’s efficiency, we analysed five specimens of European eel as model hosts. Their parasites were identified morphologically, and then all the parasites and the entire host tissue were homogenised together using a commercial blender. Molecular identification of the morphologically detected parasites was conducted using DNA barcoding with parasite-specific primers. Following homogenisation and DNA-based identification, we successfully detected all parasite taxa identified during morphological analyses, even including instances where they had not been detected morphologically. A notable exception were acanthocephalans, which showed low levels of molecular detection. Despite certain limitations, the detection of parasites directly from the whole-host homogenate shows high potential for efficient and accurate parasite detection, in some cases even surpassing morphological identification. The further development of the method, particularly through exploration of DNA metabarcoding, could improve the reliability of parasite assessments and facilitate parasite detection, which could aid in proper parasite recognition.

## Highlights

• A new approach for DNA-based parasite detection using whole-host homogenisation.
• The new approach showed high congruence with parasite identification based on morphology.
• The new approach at times outperformed the morphological methods.
• When optimised, the new approach can allow for a holistic parasite scan with DNA metabarcoding.
• The new approach can significantly reduce dependence on taxonomic expertise.

## Graphical abstract

**Figure:**
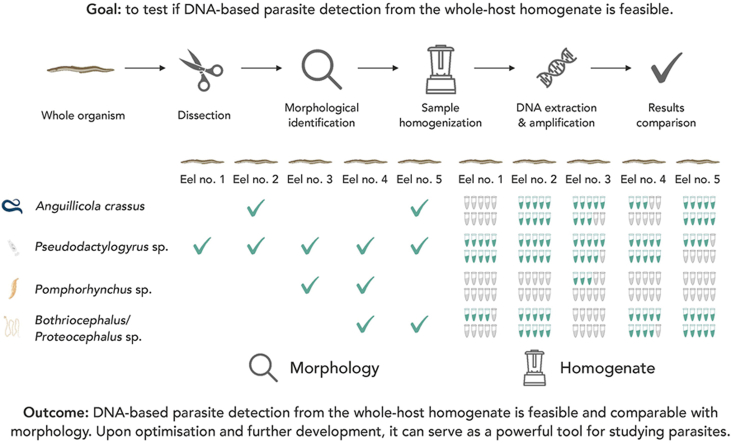

## 1 Introduction

Despite constituting an overwhelming proportion of current biodiversity and despite their importance for ecosystem functioning (Hudson et al., 2006; Dobson et al., 2008; Smit and Sures, 2025), parasites remain among the most neglected and least studied components of biodiversity. This could be due to several methodological difficulties that hamper the detection and monitoring of parasites. Morphological analyses require a substantial amount of time and require specialised taxonomic expertise for detecting all parasite life stages and correct species identification. Also, given minute morphological differences among closely related parasite taxa and the fragility of the parasite tissues, morphology-based parasite identification is prone to error (Bruno et al., 2006; Perkins et al., 2011). Therefore, to mitigate these limitations, molecular approaches involving DNA-based parasite identification, primarily based on DNA barcoding, have been proposed (Perkins et al., 2011; Blasco-Costa et al., 2016) and are now commonly used for parasite identification, often in parallel to morphological identification. However, finding and retrieving the parasite tissue for molecular analyses still requires a certain level of specialised knowledge. Given the ongoing taxonomic impediment (Poulin et al., 2020), new approaches providing reliable parasite identification are welcome.

One of the strategies for dealing with biological samples containing multiple taxa is sample homogenisation. This method has already been tested for processing bulk samples derived from Malaise traps or macroinvertebrate kick-net samples using DNA metabarcoding. Sample processing protocols involving sample homogenisation often outperformed other methods in terms of retrieved species diversity. At the same time, it reduces sample processing time by excluding the need for sample sorting, showing a great promise for studying biodiversity (Buchner et al., 2021; Pereira-da-Conceicoa et al., 2021; Zizka et al., 2022). If one considers an individual organism as a bulk sample serving as a host to a variety of organisms, homogenisation seems also a viable option for studying its parasite biodiversity. However, to our knowledge, this approach has so far never been used for studying parasites, although in many cases, homogenisation of the entire host is feasible even with commercially available devices.

Therefore, the goal of this study was to test whether DNA-based parasite detection and identification based on whole-host homogenisation is feasible and equally effective in terms of detection efficiency compared to morphological parasite examination. With that, our study serves as a proof of concept for the subsequent method development for holistic parasite surveys through DNA metabarcoding. This in turn, could not only serve as a complementary tool for morphological identification but also would allow studying parasite composition without extensive taxonomic expertise.

## 2 Materials and methods

### 2.1 Material collection and maintenance

As host model, we used five adult specimens of European eels (*Anguilla anguilla*) obtained on the 25th of September 2024 from a commercial fisherman from Grieth, Germany. European eels serve as an important fish model, being in recent years in decline due to the introduction of several parasite species that have detrimental effects on local eel populations (Sures and Knopf, 2004). After collection, eels were transported back to the aquarium facility at the University of Duisburg-Essen and kept overnight in a 500-l tank filled with tap water.

### 2.2 Morphological analyses

On the following day, all five eels (later referred to as eel no. 1–5, respectively) were euthanised and subsequently dissected during the parasitological practical course classes by the students with the help of the course supervisors. After the dissections, the parasite identification was conducted by taxonomic experts by inspecting selected organs (eyes, gills, stomachs, intestines and swim bladders) by stereomicroscopy for parasite infections with helminths used as model parasites for this study. The parasites found were then morphologically identified to the lowest possible taxonomic level.

### 2.3 Sample homogenisation

Following the morphological identification, the entire fish, including all the dissected organs and all retrieved parasites, was then placed together in commercial blenders (Shardor OK1310 model; Telos Trading Ltd, Herefordshire, UK) and homogenised at maximum speed of 24,000 *rpm* for 3 min together with 600 ml of ice-cold 100% ethanol to minimise the heating of the sample. To minimise the risk of cross-contamination, each fish was processed in a separate blender. Moreover, prior to each homogenisation, each mixing container was filled with 250 ml of self-made decontamination solution (0.6% bleach, 1% NaOH, 1% Alconox, 90 mM sodium bicarbonate) and the blender was run for 3 min. Afterwards, the container was thoroughly cleaned with ddH_2_O until no bubbles were visible. After each homogenisation, 10 extraction replicates were taken from each sample using a spoonful of a stainless-steel micro-spoon (approximately 0.5 ml per replicate).

### 2.4 DNA extraction, amplification and sequencing

Full step-by-step DNA extraction protocol and all details related to primer choice and PCR conditions are described in Supplementary file S1. In brief, the parasites were identified through DNA barcoding using primers based on the morphological identification by choosing parasite species-specific primers (Table 1). In the end, the detection of the parasite was only considered positive where a DNA sequence had been generated and identified as a target parasite taxon. All generated sequences were deposited in the NCBI GenBank database (PX635314-PX635356; PX677912-PX677945; PX684372-PX684407; see Supplementary file 2: Table S1 for details). Additionally, all sequences were compiled in the dataset and deposited in the public repository of the Barcode of Life Data Systems (BOLD) (Ratnasingham and Hebert, 2007), where all of the relevant metadata information and sequence trace files are available (project code: SMPAR).

**Table 1:** Primers used in this study.

Target organism	Target gene	Primer name	Primer sequences (5′-3′)	Reference
*Anguilla anguilla*	*cox*1	MiEel-F	CTTACAGCAAACCTGACAGCAG	Takeuchi et al. (2019)
		MiEel-R	TTGGTGTGCCATTATACGTTTTCTTG	
*Anguillicola crassus*	*cox*1	crasscox F	CCTTTTGTTAGGTGATGGGCAA	Grabner et al. (2012)
		crasscox R	TAGCGAGATCAACACTTATACCAG	
*Pseudodactylogyrus* sp.	18S rRNA	PD_18S_fwd	GTCGTAACAAGGTTTCCGTAGG	Kania et al. (2010)
		PD_18S_rev	CAAGACGGGTCTGGTGGAAC	
*Pomphorhynchus* sp.	*cox*1	PT/PL-fwd	ATGGGGTTTTCTATAAGRCTA	Tierney et al. (2020)
		PT/PL-rev	CAAATTACGATCCATCAAAAGCA	
*Bothriocephalus* sp.	*cox*1	PBI- cox1F	CATTTTGCTGCCGGTCARCAYATGTTYTGRTTTTTTGG	Scholz et al. (2013)
		PBI- cox1R	CCTTTGTCGATACTGCCAAARTAATGCATDGGRAA	
*Proteocephalus* sp.	*cox*1	B2_Trp_F	TAGACTAARTGTTTTCAAAAC	de Chambrier et al. (2019)
		B3_16S_R	GCAAAAGGCAARCAAACCTA	
Cestoda	12S rRNA	Cestode_1_F	TTAAGCYAAGTCTATGTGCTGC	Hentati et al. (2025)
		Cestode_1_R	GCGGTGTGTACMTGAGCTAAAC	

## 3 Results and discussion

### 3.1 Morphological identification

**Fig. 1:**
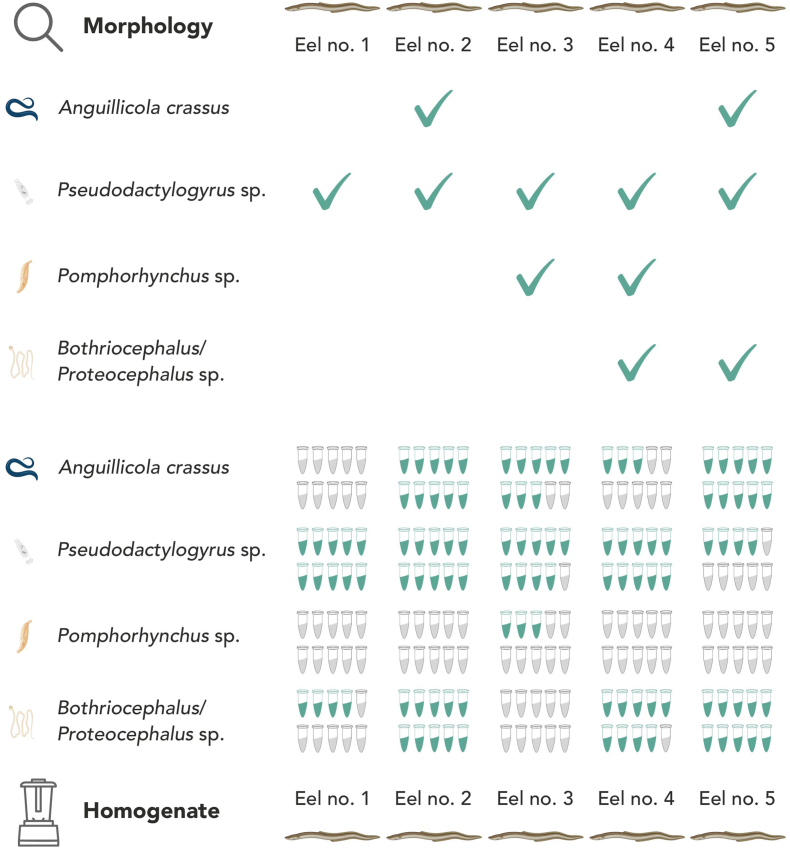
Overview of the morphological and DNA-based parasite identification results. The green ticks in the morphology section indicate positive morphological detection of a given parasite taxon. The tubes in the homogenate section refer to ten replicates used in our study for each homogenised host. The number of green tubes reflects the number of replicates that showed positive DNA-based detection based on the DNA sequencing outcome. The order of the tubes does not reflect the order of the specific replicates.

The morphological examination revealed that all five eels were infected by at least one parasite taxon (Fig. 1). Only parasite presence/absence was determined without consistent estimates of parasite intensity. Four helminth taxa were identified belonging to Nematoda (*Anguillicola crassus*; present in eels no. 2 and no. 5), Monogenea (*Pseudodactylogyrus* sp.; present in all five eels), Acanthocephala (*Pomphorhynchus* sp.; present in eels no. 3 and no. 4) and Cestoda (*Bothriocephalus*/*Proteocephalus* sp.; present in eels no. 4 and no. 5). In case of cestodes, the uncertainty in terms of taxonomic assignment derives from the lack of gravid proglottids and the scolex, which serve as key taxonomic features (Scholz et al., 1998; Oros et al., 2010). In that case, the taxonomic identification was narrowed down to *Bothriocephalus*/*Proteocephalus* sp., the only cestodes known from eel from this area (Sures and Streit, 2001). This case illustrates one of several difficulties associated with morphology-based identification of the fish parasites, which heavily relies on the completeness of the studied specimens as well as their proper sampling and preservation (Kvach et al., 2018).

### 3.2 Molecular identification based on whole-host homogenates

All samples were positive for eel DNA, confirming the success of the DNA extractions (Supplementary file 3: Fig. S1). Detailed information about individual replicates and their gel and sequencing performance is provided in Supplementary file 2: Table S1 and Supplementary file 3: Figs. S2–S5. Regarding taxonomic identification of the newly generated parasite sequences, all sequences obtained using *A. crassus*-specific primers represented *A*. *crassus* at 100% similarity to the reference data. All *Pseudodactylogyrus* sp. sequences showed 99.9% similarity to *Pseudodactylogyrus bini*. For *Pomphorhynchus* sp. detection, all sequences showed 100% similarity to *Pomphorhynchus bosniacus*. For cestode sequences, given a short sequence fragment length (approximately 100 bp), the verification through the BLAST function was not possible. Therefore, a subset of 12S rRNA gene sequences was mined from NCBI GenBank manually and subsequently aligned. This alignment, as well as the *Bothriocephalus claviceps* samples that were amplified as positive controls for cestode primers (GenBank: PX684405-PX684407), served as a basis for the taxonomic identification of the obtained cestode sequences. The sequences obtained were grouped into three distinct units based on their genetic distance: the first group encompassed sequences from eel no. 1; the second group comprised sequences from eel no. 4; and the final group consisted of sequences from eels no. 2 and no. 5. The first two groups were generally similar, sharing 94.5% similarity. Both were distinctly different from the other group, ranging from 75.8 to 73.6% of similarity. Based on the similarity to mined sequences and the positive controls, the first two groups showed the highest similarity to *Bothriocephalus* sp., with sequences from eel no. 4 being 100% identical to the *B*. *claviceps* positive control. These two groups also showed the highest similarity to members of the family Bothriocephalidae present in the NCBI GenBank database, with the first group showing the highest similarity (at 81.3%) to *Senga ophiocephalina* (GenBank: KX434430 and NC034715) and the second group, including *B. claviceps* samples, showed the highest similarity to *Schyzocotyle acheilognathi* (GenBank: KX589243) at 79.1% similarity. Regarding the final group, the closest available matches were members of the family Rhinebothriidae (e.g. GenBank: MZ594613) at 75.1% similarity.

### 3.3 Comparability between morphological and DNA-based identification

Regarding comparability between morphological and molecular *A. crassus* identification, positive detections were observed for eel no. 2 (10 out of 10 replicates), eel no. 3 (8 out of 10 replicates), eel no. 4 (3 out of 10 replicates) and eel no. 5 (10 out of 10 replicates), while no *A. crassus* was detected in any replicate for eel no. 1 (Fig. 1). The results show congruence with morphological identification. Interestingly though, the parasite signal was also detected from two specimens where no adult nematodes were observed. On one hand, this might indicate possible contamination; however, given all the measures taken, this seems unlikely. On the contrary, the observed results may suggest an early stage of infection, with third-stage larvae migrating through the eels or residing in the swim bladder wall. For example, migrating third-stage larvae (100–300 μm) en route to the swim bladder have never been observed so far. Accordingly, larval infection might not be visible to the human eye, although it can already be detectable through DNA-based identification from the fish homogenate. In these instances, the proposed approach outperforms a morphology-based detection of parasites.

For *Pseudodactylogyrus* sp., positive detections were observed for all eels, ranging from 10 out of 10 replicates (for eels no. 1, no. 2 and no. 4), 9 out of 10 replicates (for eel no. 3), and 3 out of 10 replicates (for eel no. 5). Here, results also show high congruence between morphological and DNA-based identification. The low detection rate observed in one of the specimens might be related to the relatively low abundance of monogeneans in the studied specimen.

A similar reason could explain observed patterns in *Pomphorhynchus* sp. Here, positive detections were only observed for eel no. 3 (3 out of 10 replicates). Regarding eel no. 4, although *Pomphorhynchus* sp. has been observed morphologically, no positive detection on a molecular level was observed. The most plausible explanation for the low detection success could be low parasite biomass; however, since the parasite abundance was not evaluated during morphological examination, it remains speculative. At the same time, DNA degradation due to the homogenisation might also explain the relatively low success observed for acanthocephalans. Therefore, the obtained results support the need for using increased replication, particularly when parasite size and/or biomass is expected to be low. Based on the results of our study, using at least five replicates per sample seems necessary.

For *Bothriocephalus*/*Proteocephalus* sp., none of the replicates showed a visible band, neither for *Bothriocephalus-* nor *Proteocephalus-*specific primers, despite the fact that all positive controls applied showed a positive amplification, but only for *Bothriocephalus*-specific primers. For more general primers targeting shorter DNA fragments, the positive detection was confirmed for eels no. 1 (4 out of 10 replicates), eel no. 2 (10 out of 10 replicates), eel no. 4 (9 out of 10 replicates) and eel no. 5 (10 out of 10 replicates). That either indicates that we are dealing with different taxa than initially expected or that the DNA in fish homogenates is degraded and cannot be amplified for longer DNA fragments.

With shorter fragments, we observed positive detections and not only in eels where we observed cestodes morphologically, but also in two other eels where no cestode was found during morphological analyses, similarly to *A. crassus*. Even though we detected members of the genus *Bothriocephalus* in two of the positive eels*,* we were only able to confirm it by comparison to the sequences of the positive control used (DNA extract of morphologically identified *Bothriocephalus* specimen), since publicly available data on 12S only allow for reliable taxonomic identification of a few cestode taxa at the moment. Due to that, the taxonomic identity of the cestode taxon observed in the other two eels remains unresolved. These results further support the possibility of DNA degradation taking place during homogenisation and support the need for filling up the gaps in public DNA repositories for parasite taxa.

### 3.4 Limitations

Although the results showed high congruence between morphological and whole-host homogenate detection of parasites, the proposed approach has important ecological, methodological and practical limitations. Detecting parasites through whole-host homogenate cannot provide information regarding developmental stage and anatomical location, which are essential for linking transmission pathways and ecological impacts across trophic levels. Furthermore, molecular identification cannot provide any reliable estimates of parasite abundances and resulting parasite intensity. Therefore, for detailed parasitological examinations aiming at studying parasite taxonomy, ecology and life cycles, the approach should only be used in combination with morphological examination, serving as a complementary cross-validation tool. Moreover, the molecular identification relies on the completeness of reference databases which are still far from complete for most of the parasites. Finally, while the method is in principle applicable to any host-parasite system, using commercial blending machines imposes practical limitations for larger hosts (e.g. marine mammals). In such cases, the protocol could be adapted to target specific organs of interest rather than processing the entire host.

### 3.5 Future directions

In the current state, the proposed approach offers only limited insight into parasite diversity by focusing on selected taxa through DNA barcoding. The natural next step should be testing DNA metabarcoding using a set of more general primer pairs for a holistic parasite scan. For that, novel primer pairs would have to be designed to appropriately cover the target parasite groups. If multiple primer pairs are needed, depending on the scope of the study, those primers ideally could be multiplexed to minimise time and cost of the laboratory procedures (Markoulatos et al., 2002). When fully optimised and validated, this improved approach will provide a robust and reliable tool for parasite detection and identification, significantly reducing dependence on taxonomic expertise. With that, the approach could serve as a more general way to verify if the host was parasitised while providing also insights into parasite diversity. However, the taxonomic assignment strongly relies on the completeness of the reference databases and therefore, future efforts should also focus on filling up the gaps for the parasite taxa while ensuring the record accuracy through entry curation with taxonomic experts. At the same time, we acknowledge that the proposed approach still requires sacrificing the hosts to detect their parasite communities, which in times of the current biodiversity crisis is still far from ideal. Therefore, the efforts of the scientific community should be placed on developing non-invasive parasite detection methods, e.g. through environmental DNA (Hupało et al., 2025). Until then, the approach proposed in this paper provides a promising starting point for improving the process of parasite detection.

## 4 Conclusion

The present results provide proof of concept that DNA-based detection of parasites from the whole-host homogenate is feasible. When further developed and optimised, the proposed approach can serve as an important and reliable tool for the parasite detection while also mitigating for ongoing taxonomic impediment and complement current morphology-based parasite surveys.

## Ethical approval

The study was carried out in accordance with the relevant guidelines and regulations and was approved by the Ethics Council (Landesamt für Natur, Umwelt und Verbraucherschutz, Nordrhein-Westfalen, permit number: 81–02.05.40.21.061).

## CRediT authorship contribution statement

**Kamil Hupało:** Conceptualisation, Data curation, Formal analysis, Investigation, Methodology, Supervision, Visualisation, Writing – original draft, Writing – review & editing. **Celine Sassor:** Investigation, Visualisation, Writing – original draft, Writing – review & editing. **Mia Merle Fuhren:** Investigation, Writing – review & editing. **Dominik Buchner:** Conceptualisation, Investigation, Methodology, Writing – review & editing. **Daniel Grabner:** Conceptualisation, Investigation, Supervision, Writing – original draft, Writing – review & editing. **Bernd Sures:** Conceptualisation, Supervision, Funding acquisition, Writing – original draft, Writing – review & editing.

## Statement on the use of AI-assisted technologies

We used OpenAI’s ChatGPT to assist with improving the clarity and grammar of the manuscript. All content was reviewed and edited by the authors. The authors take full responsibility for the content of the published article.

## Funding

This research was funded by a Biodiversa+ project ‘Integrated Monitoring of Parasites in Changing Environments (IMPACT)’ co-funded by the 10.13039/501100000780European Commission (GA No. 101052342) and the German Research Foundation (DFG, SU 217/23-1, project no. 532239906).

## Declaration of competing interests

The authors declare that they have no known competing financial interests or personal relationships that could have appeared to influence the work reported in this paper.

## Data Availability

The data supporting the conclusions of this article are included within the article and its supplementary files. All newly generated sequences were deposited in the NCBI GenBank database under the following accession numbers: PX635314-PX635356; PX677912-PX677945; PX684372-PX684407. Additionally, all sequences were compiled in the dataset and deposited in the public repository of the Barcode of Life Data Systems (BOLD), where all of the relevant metadata information and sequence trace files are available (project code: SMPAR).

## Appendix ASupplementary data

Supplementary file 1 **Text S1.** Detailed description of PCR methods used in this study. Supplementary file 1

Supplementary file 2 Table S1**.** List of all individual replicates and their gel and sequencing performance grouped by target parasite taxa. Supplementary file 2

Supplementary file 3 Fig. S1**.** Annotated results of gel electrophoresis for European eel-specific primers serving as a positive control for DNA extraction success. Fig. S2. Annotated results of gel electrophoresis for *Anguillicola crassus*. Fig. S3. Annotated results of gel electrophoresis for *Pseudodactylogyrus* sp. Fig. S4. Annotated results of gel electrophoresis for *Pomphorhynchus* sp. Fig. S5. Annotated results of gel electrophoresis for cestodes. Supplementary file 3
