# Supplementary material for: Assessing whole-host homogenisation as a new tool for parasite detection and identification
Source: Curr Res Parasitol Vector Borne Dis. 2026 Jan 1;9:100348. doi: 10.1016/j.crpvbd.2026.100348 (PMC12818088; doi:10.1016/j.crpvbd.2026.100348)
Supplement: Supplementary file 1 — Text S1. Detailed description of PCR methods used in this study. [file mmc1.pdf]

Supplementary file S1 – details of DNA extraction, amplification and sequencing

DNA extraction

DNA extraction for all 10 replicates per each sample were done using an automated liquid handling robot (Hamilton Vantage, Hamilton Bonaduz AG, Switzerland). A full step-by-step sample lysis protocol has been deposited on the protocols.io repository (dx.doi.org/10.17504/protocols.io.dm6gpjrmjgzp/v1). From the resulting lysate, 300 µL were transferred to a 2.2 mL deepwell plate and used for subsequent DNA extraction. DNA extraction was performed using a positive pressure manifold (MPE2, Hamilton, Bonaduz, Switzerland) using a slightly modified, silica-column-based version of the protocol deposited on the protocols.io repository (dx.doi.org/10.17504/protocols.io.eq2ly73mmlx9/v2) which involved an alternate binding buffer (2.5 M GuHCl, 120 mM NaAc, 80% EtOH, pH 5), resulting in an elution volume of 100 µL. Extraction success was confirmed through gel electrophoresis using 1% agarose gel. The extracted samples from one of the technical replicates were then subjected to PCR amplification.

Primer choice and DNA amplification

The extracted samples from one of the technical replicates were then subjected to PCR amplification. First, to verify if the DNA extraction worked, the primers specific to *Anguilla anguilla* (Grabner et al., 2012) were used using 5 µL of Accustart II Supermix (2x) (QuantaBio, Beverly, MA, USA), 1 µL of each primer (2 µM), 2 µL of PCR water and 1 µL of DNA extract. For amplification, the following protocol was used: initial denaturation at 95°C for 5 min followed by 40 cycles of denaturation at 95°C for 30 s, annealing at 65°C for 90 s and extension at 72°C for 30 s with a final elongation at 68°C for 10 min.

For the parasite infection testing, the primer choice was based on the results of morphology-based taxonomic assignment and included using species-specific primer pairs for each parasite taxon identified, i.e. *Anguillicola crassus*, *Pseudodactylogyrus* sp., *Pomphorhynchus* sp., *Bothriocephalus/Proteocephalus* sp. (Takeuchi et al., 2019, Grabner et al., 2012, Kania et al., 2010, Scholz et al., 2013, Uhrová, 2020, Tierney et al., 2020, Hentati et al., 2025) (Table 1). In case of *Bothriocephalus/Proteocephalus* sp., the more genus-specific primers used initially did not show any amplification success. Therefore, a third, more general primer pair amplifying a short fragment of 12S was used.

The PCR setup for specific primers was as follows: for *Anguillicola crassus*: 5 µl of Accustart II Supermix (2x) (QuantaBio), 1 µl of each primer (2 µM), 2 µl of PCR-grade water and 1 µl of DNA extract. The PCR conditions were as follows: initial denaturation at 95°C for 5 min followed by 40 cycles of denaturation at 95°C for 30 s, annealing at 65°C for 90 s and extension at 72°C for 30 s with a final elongation at 68°C for 10 min. For *Pseudodactylogyrus* sp.: 10 µl of DreamTaq Master Mix (2x) (Thermo Fisher), 1 µl of each primer (2 µM), 6 µl of PCR-grade water and 2 µl of DNA extract. The PCR conditions were as follows: initial denaturation at 95°C for 3 min followed by 40 cycles of denaturation at 95°C for 30 s, annealing at 52°C for 90 s and extension at 72°C for 120 s with a final elongation at 72°C for 10 min. For *Pomphorhynchus* sp.: 10 µl of DreamTaq Master Mix (2x) (Thermo Fisher), 1 µl of each primer (2 µM), 6 µl of PCR-grade water and 2 µl of DNA extract. The PCR conditions were as follows: initial denaturation at 95°C for 5 min followed by 35 cycles of denaturation at 95°C for 30 s, annealing at 58°C for 90 s and extension at 72°C for 30 s with a final elongation at 72°C for 10 min. For *Bothriocephalus* sp.: 10 µl of DreamTaq Master Mix (2x) (Thermo Fisher), 1 µl of each primer (2 µM), 6 µl of PCR-grade water and 2 µl of DNA extract. The PCR conditions were as follows: initial denaturation at 95°C for 3 min followed by 35 cycles of denaturation at 95°C for 30 s, annealing at 60°C for 90 s and extension at 72°C for 60 s with a final elongation at 72°C for 10 min. For *Proteocephalus* sp.: 10 µl of DreamTaq Master Mix (2x) (Thermo Fisher), 1 µl of each primer (2 µM), 6 µl of PCR-grade water and 2 µl of DNA extract. The PCR conditions were as follows: initial denaturation at 95°C for 3 min followed by 35 cycles of denaturation at 95°C for 30 s, annealing at 56°C for 90 s and extension at 72°C for 120 s with a final elongation at 72°C for 10 min. For cestode primers: 10 µl of DreamTaq Master Mix (2x) (Thermo Fisher), 1 µl of each primer (2 µM), 6 µl of PCR-grade water and 2

µl of DNA extract. The PCR conditions were as follows: initial denaturation at 95°C for 3 min followed by 10 cycles consisting of denaturation at 95°C for 30 s, annealing at 57°C for 30 s with a decrement of 0.5°C per cycle, and extension at 72°C for 60 s. Subsequently, 35 additional cycles were performed with denaturation at 95°C for 30 s, annealing at 52°C for 30 s, and extension at 72°C for 60 s with a final elongation at 72°C for 10 min.

Apart from samples from the fish homogenates, single PCR negative control as well as a set of positive controls, being DNA extracts from individuals of respective parasite species (except for *Proteocephalus* sp.) were used to verify the amplification success. The parasite extracts derive from individual parasite specimens either collected during the course of this study (two individuals of *A. crassus*) or collected from the eel hosts deriving from the same wild population as used in this study, but dissected in April 2024 and used for a different study (unpublished data). Those included two extracts of *A. crassus* (extracted during this study following the procedure described above), three extracts from *Pseudodactylogyrus* sp., three extracts from *Pomphorhynchus* sp. and three extracts from *Bothriocephalus claviceps*, which were used for respective PCRs.
To increase the reliability of the obtained results, the PCRs were run in duplicates and the amplification success was verified through gel electrophoresis (all annotated gels presented in supplementary figures S1-S5). All the individuals which showed positive detection at least in one of the replicates were subjected to subsequent Sanger sequencing. Generally, the positive controls were not subjected to sequencing except for Cestoda, given the novelty of the primers. Before sequencing, 5 µL of the PCR products were purified enzymatically with 10 U of Exonuclease I (ExoI) and 1 U of thermosensitive alkaline phosphatase (FastAP) (both Thermo Fisher Scientific) with an incubation step at 37°C for 15 min followed by an inactivation step at 85°C for 15 min. Unidirectional Sanger sequencing of the forward direction (using respective forward primers) was performed at Eurofins Genomics (Cologne, Germany). For individuals where the sequencing did not provide usable DNA sequences, one round of reamplification and subsequent resequencing was performed using the same protocol (all individuals listed in Supplementary file 2: Table S1). All generated sequences were deposited in NCBI GenBank (PX635314-PX635356; PX677912-PX677945; PX684372- PX684407). Additionally, all sequences were compiled in the dataset and deposited in the public repository of the Barcode of Life Data Systems (Ratnasingham and Hebert, 2007)), where all of the relevant metadata information and sequence trace files are available (project code: SMPAR).

Sequence data analyses and parasite detection
The quality of the obtained sequences was checked in Geneious Prime 2025.0.2 (https://www.geneious.com) and aligned with MAFFT 1.4.0 (Katoh and Standley, 2013) as implemented in Geneious using the automatic algorithm selection and default settings. The taxonomic assignment of the sequences was done using the BLAST function implemented in the Geneious software, which is based on DNA sequence information deposited in the NCBI GenBank database.
