# Supplementary material for: Assessing whole-host homogenisation as a new tool for parasite detection and identification
Source: Curr Res Parasitol Vector Borne Dis. 2026 Jan 1;9:100348. doi: 10.1016/j.crpvbd.2026.100348 (PMC12818088; doi:10.1016/j.crpvbd.2026.100348)
Supplement: Supplementary file 3 — Fig. S1. Annotated results of gel electrophoresis for European eel-specific primers serving as a positive control for DNA extraction success. Fig. S2. Annotated results of gel electrophoresis for Anguillicola crassus. Fig. S3. Annotated results of gel electrophoresis for Pseudodactylogyrus sp. Fig. S4. Annotated results of gel electrophoresis for Pomphorhynchus sp. Fig. S5. Annotated results of gel electrophoresis for cestodes. [file mmc3.pdf]

Supplementary file 3. Annotated results of gel electrophoresis for all primer pairs.

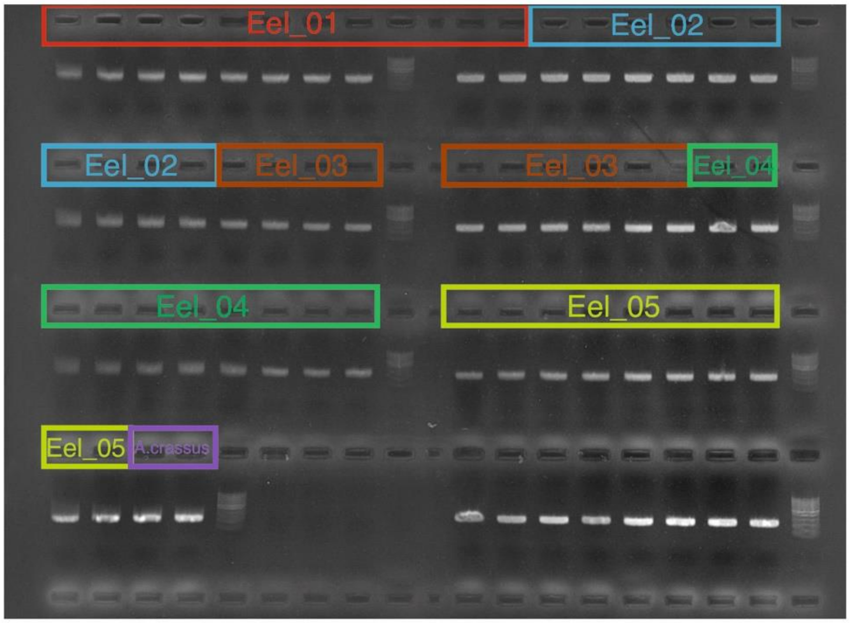

Supplementary Figure S1. Annotated results of gel electrophoresis for European eel-specific primers serving as a positive control for DNA extraction success.

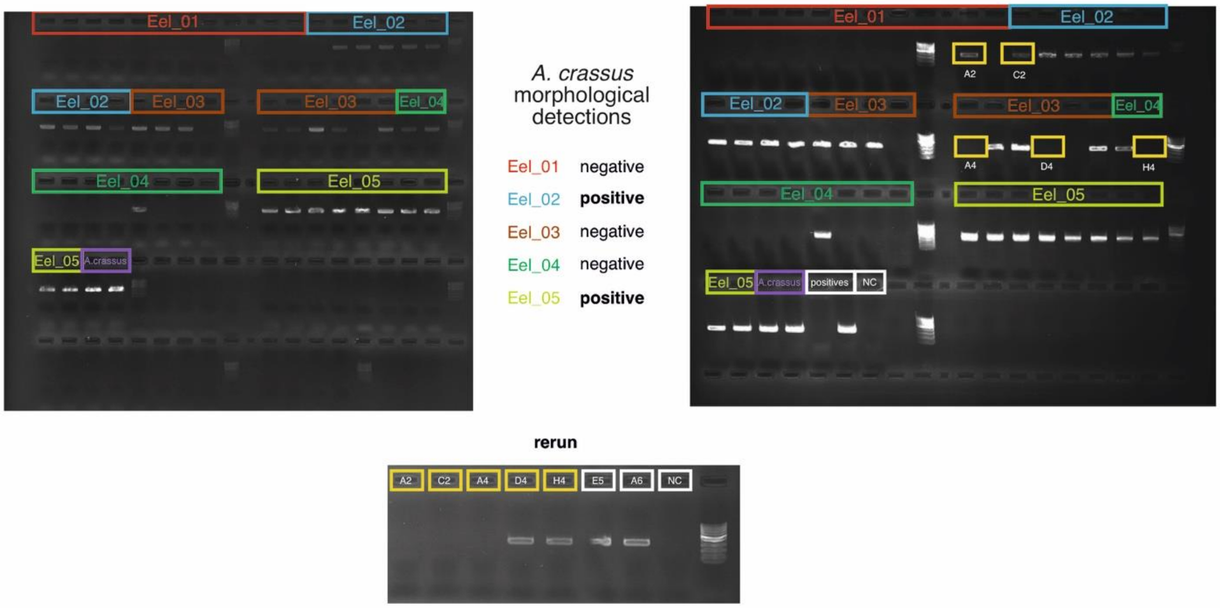

Supplementary Figure S2. Annotated results of gel electrophoresis for *Anguillicola crassus*.

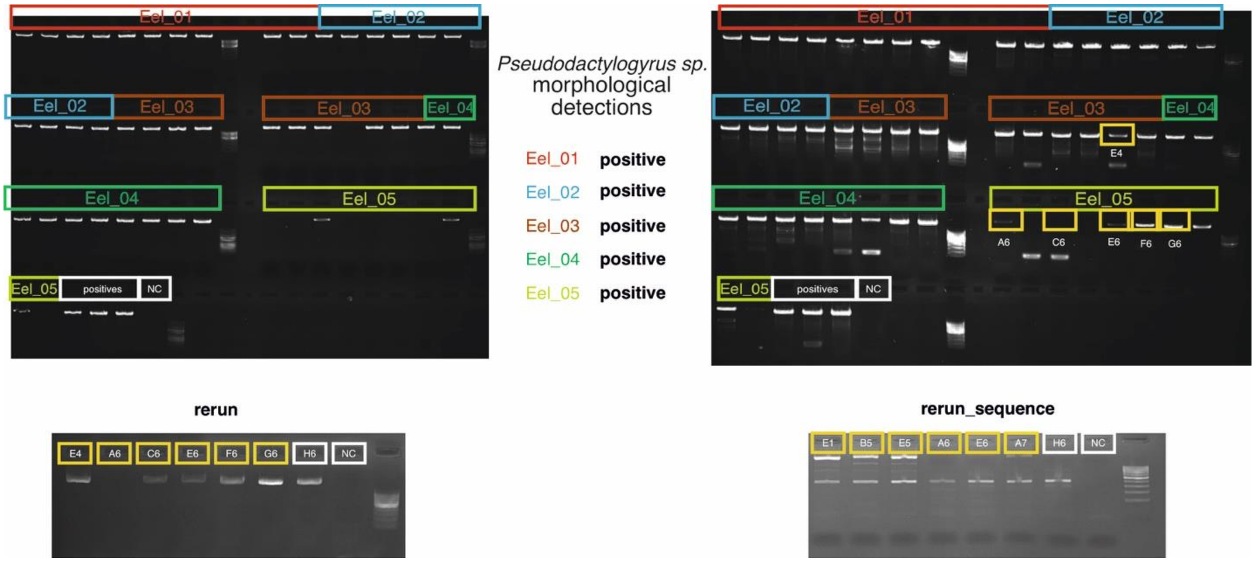

Supplementary Figure S3. Annotated results of gel electrophoresis for *Pseudodactylogyrus* sp.

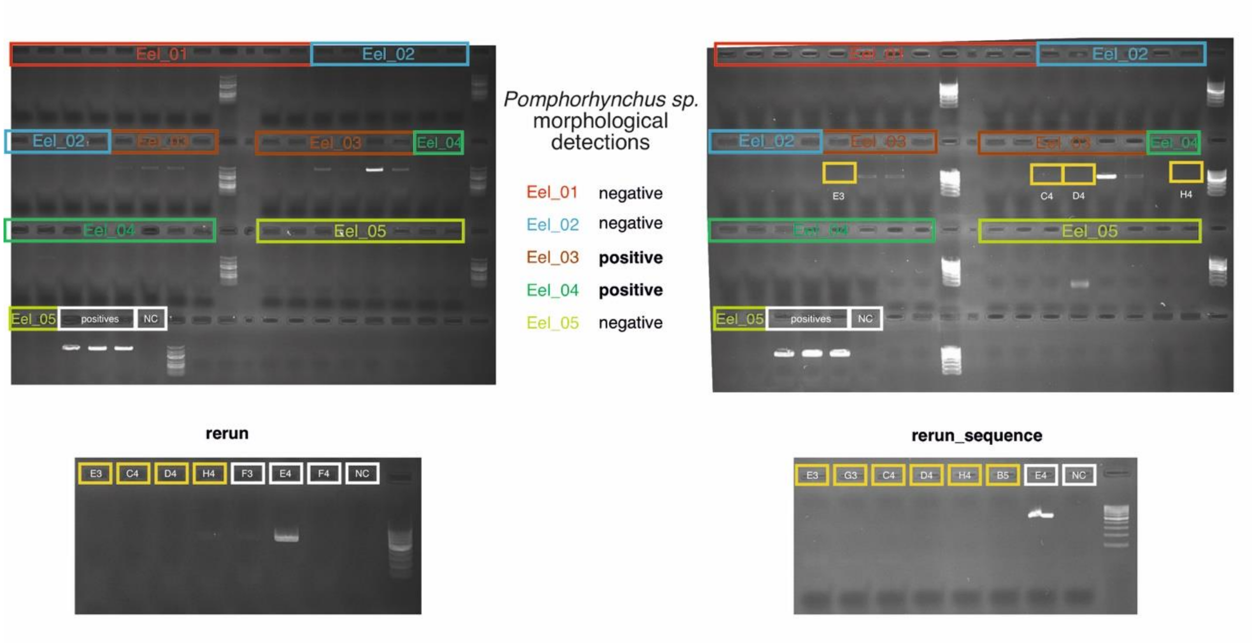

Supplementary Figure S4. Annotated results of gel electrophoresis for *Pomphorhynchus* sp.

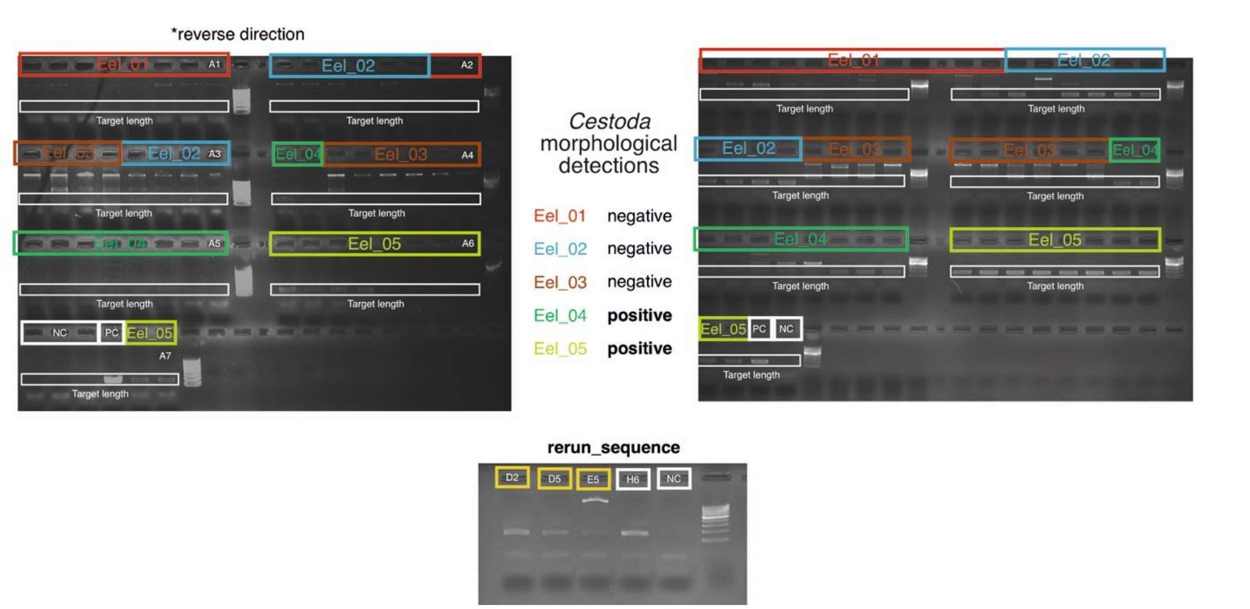

Supplementary Figure S5. Annotated results of gel electrophoresis for cestodes.
